# Self-Shielding Analysis of the Zap-X System

**DOI:** 10.7759/cureus.1917

**Published:** 2017-12-06

**Authors:** Georg A. Weidlich, M. Bret Schneider, John R. Adler

**Affiliations:** 1 Radiation Oncology, National Medical Physics and Dosimetry Company, Palo Alto, USA; 2 Department of Neurosurgery, Stanford University School of Medicine, California, USA; 3 Department of Radiation Oncology, Stanford University Medical Center, Stanford, CA, USA

**Keywords:** self-shielded, radiation, stereotactic radiosurgery, radiation exposure, radiation shielding

## Abstract

The Zap-X is a self-contained and first-of-its-kind self-shielded therapeutic radiation device dedicated to brain as well as head and neck stereotactic radiosurgery (SRS). By utilizing an S-band linear accelerator (linac) with a 2.7 megavolt (MV) accelerating potential and incorporating radiation-shielded mechanical structures, the Zap-X does not typically require a radiation bunker, thereby saving SRS facilities considerable cost. At the same time, the self-shielded features of the Zap-X are designed for more consistency of radiation protection, reducing the risk to radiation workers and others potentially exposed from a poorly designed or constructed radiotherapy vault. The hypothesis of the present study is that a radiosurgical system can be self-shielded such that it produces radiation exposure levels deemed safe to the public while operating under a full clinical workload. This study summarizes the Zap-X system shielding and found that the overall system radiation leakage values are reduced by a factor of 50 compared to the occupational radiation limit stipulated by the Nuclear Regulatory Commission (NRC) or agreement states. The goal of self-shielding is achieved under all but the most exceptional conditions for which additional room shielding or a larger restricted area in the vicinity of the Zap-X system would be required.

## Introduction

The Zap-X is a new, dedicated self-contained and self-shielded radiosurgery system developed and manufactured by ZAP Surgical Systems, Inc. of San Carlos, California. This device is intended for stereotactic radiosurgery (SRS) treatment of benign and malignant intracranial and cervical spine lesions (in some cases as inferior as C7). A 2.7 megavolt (MV) S-band linear accelerator (linac) is the source of therapeutic radiation. Akin to a large gyroscope, the linac is mounted within a combination of yoked gimbals with attached radiation shielding, each of which accurately rotates around a common isocenter. This mechanical construct enables the linac beam to crossfire from 2$\pi$ steradians of solid angle, as is ideally required for cranial SRS.

Accurate therapeutic beam positioning is accomplished through the two above-mentioned independent rotations of the accelerator, and precise movements of a robotic patient table. Most components needed to produce the beam, such as the radiofrequency power source, waveguiding system, and beam triggering electronics, as well as significant radiation shielding, are mounted on or integrated into the rotating patient treatment chamber sphere. The patient is supported on a moveable treatment table that extends outside the treatment sphere but which itself is also enclosed by additional radiation shielding during radiosurgery. This table shielding consists of a rotary shell and pneumatic door on a steel frame.

The Zap-X accomplishes precise three-dimensional (3D) patient registration by means of an integrated planar kilovolt (kV) imaging system that also rotates around the patient’s head. Pairs of non-coaxial x-ray images and image-to-image correlation are utilized to determine the location of the patient’s anatomy with respect to the machine isocenter, both prior to and during radiosurgical treatment.

The hypothesis of the present study was that a radiosurgical system can be self-shielded such that it produces radiation exposure levels deemed safe to the public by National Council on Radiation Protection (NCRP) standards, while operating under a full clinical workload. This hypothesis would be evaluated by taking direct measurements of transmitted radiation at a series of stations around the periphery of the device, and comparing these readings to these nationally accepted standards for radiation safety.

## Materials and methods

The shielded patient chamber and treatment table shell, together with a rotating beam stop, were designed by Monte Carlo simulation to provide an amount of shielding effect that, in conventional systems, would be provided by the walls, ceiling, and floor of a radiotherapy vault. The goals of this system are to:

a. Provide shielding to personnel outside a 1-meter (m) safety zone from the perimeter of the Zap-X system to levels that are acceptable to the public (1 millisievert (mSv)/year). This limit is generally applicable to radiation workers and non-radiation workers and stated by the National Council on Radiation Protection [1].

b. Provide shielding at any one point along the above-described perimeter line that results in an instantaneous exposure rate of no more than 2.0 milliroentgens in any one hour.

c. Meet the design requirement of being self-shielded.

Shielding is composed principally of iron; however, where required, there are also supplemental high Z materials consisting of steel, lead, or tungsten alloys. In terms of design, a priori knowledge of where to place radiation shielding, as well as the thickness and materials to be used, was determined using radiation transport computational simulations based on the Monte Carlo dose algorithm BEAMnrc [2-4]. The beam was modeled based on the geometry of the beam-defining components such as the X-ray target, primary and secondary collimators. The beam model was validated based on comparison to published and measured attenuation characteristics of the shielding materials used. Primary radiation, as well as leakage and scatter radiation, was taken into account, and the required treatment sphere wall thicknesses were determined at any point on this sphere and designed from iron, lead, or tungsten according to the Radiation Protection guidelines defined in NCRP reports 49, 51, 116, and 151 [1].

**Figure 1:**
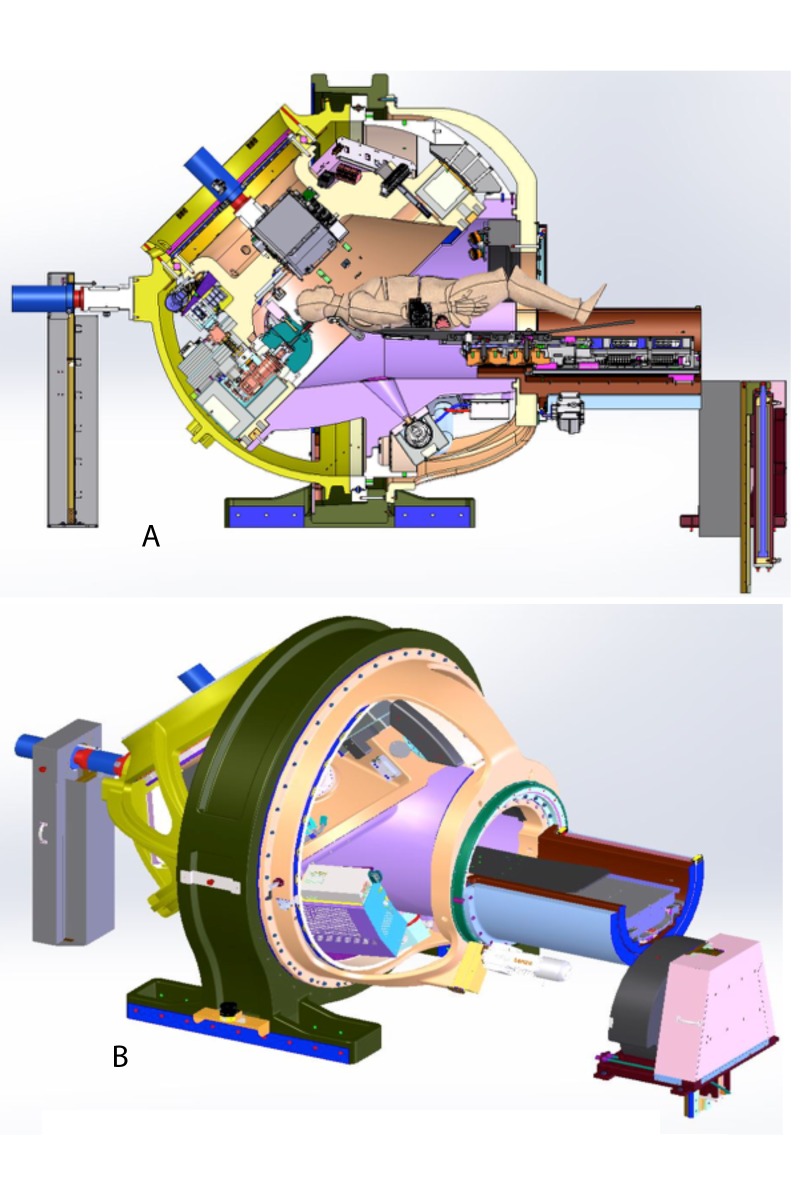
The Zap-X system and its shielding. (A) Cross-sectional view of the Zap-X system; (B) Room's eye view of the Zap-X system.

The cross-sectional view and beam’s eye view of the Zap-X are shown in Figure 1A and Figure 1B.

In the present study, the reference for interpreting test results was chosen to be the specific assumptions that went into the initial design of the Zap-X.

The primary supposition was that a fully-booked radiosurgery clinic facility would conduct nine 20 Gy isocentric treatments five days per week, 50 weeks per year, each being prescribed to the 80% isodose line and delivered with the largest 25 mm collimator. Such radiosurgical treatments were all planned on the Zap-X treatment planning system (TPS) [5], each consisting of a full set of 206 beams with an equal dose weight resulting in 6250 monitor units (MU). The above assumption results in 2250 treatments administered per year, translating into an annual workload of 1.406 x 10^7^ MU, which, in turn, represents 156.25 hrs/year of beam-on time. Utilizing a dose rate of 1500 MU/min, the duty cycle is therefore 156.25 beam hrs/2000 work hours per year = 0.078; we see that only 7.8% of the operational time the beam is on.

The MU per treatment can be calculated to be:

MU/treatment = D/(80% x PDD) = 2000 cGy/(0.8 x 0.4) = 6250 MU, where D equals dose, PDD equals percent depth dose (40%).

The total workload in MUs will be:

6250 MU/treatment x 2250 treatments/year = 1.406 x 10^7^ MU/year.

With a dose rate of 1500 MU/min, the annual beam-on time will be:

T = 1.406 x 10^7^ MU/1500 MU/min = 9373.33 min = 156.22 hrs.

The utilization factor can now be calculated to be:

156.22 hrs/year/2000 hrs/year = 0.078.

This utilization represents the maximum practical amount of MUs that can be delivered in an eight-hour period. It is anticipated that only 30-35% of treatments will have multiple isocenters. For these cases, the amount of MU's will increase, but not proportionally to the number of isocenters, as fewer beams per isocenter will be used. The number of treatments per day will decrease proportionally to the increase in MU's per treatment used. The annual workload of 1.406 x 10^7^ MU is not expected to be exceeded except for extremely busy treatment schedules.

Radiation shielding as a function of beam position

**Figure 2:**
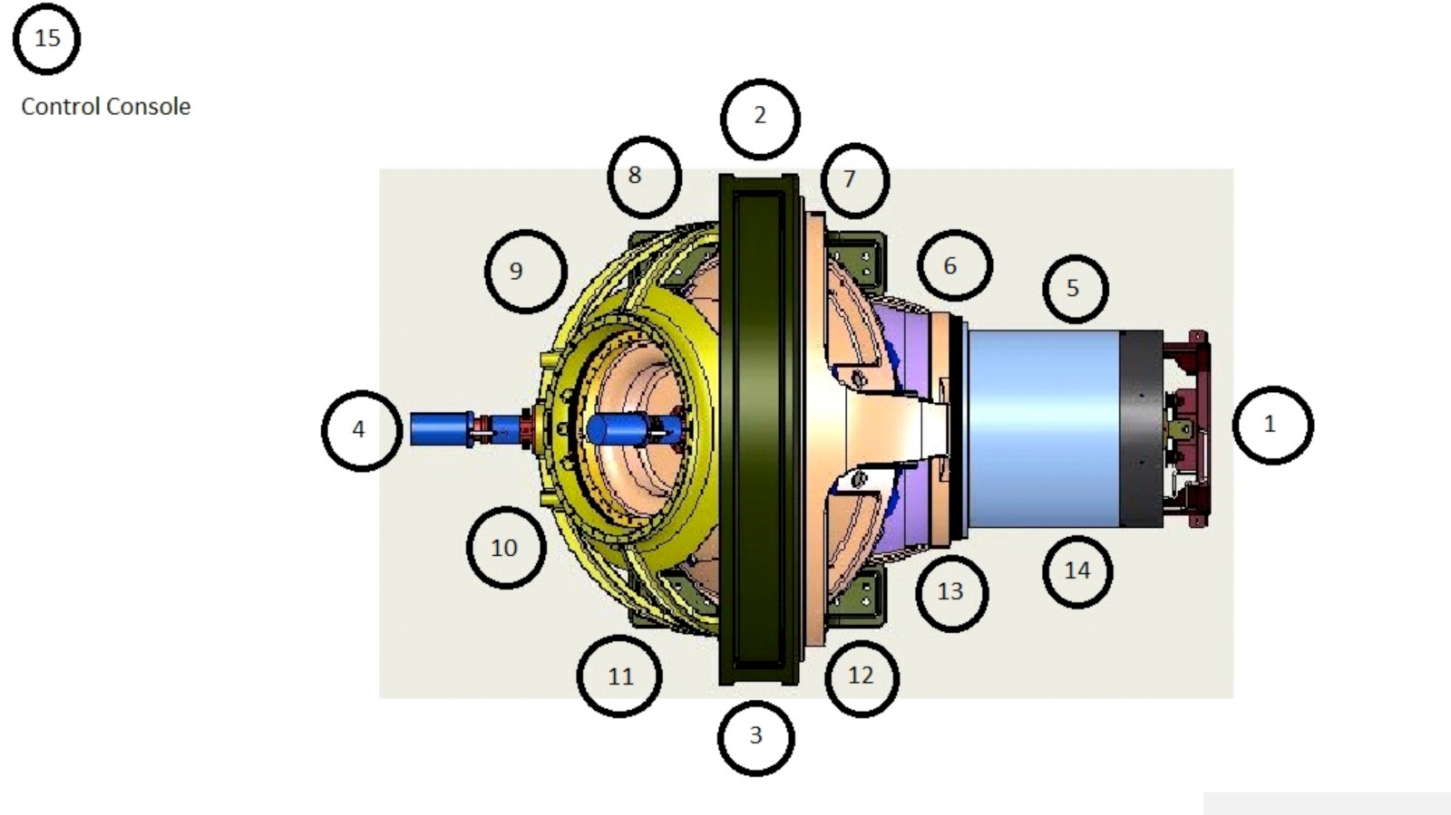
Summary of measurement stations.

Access to the Zap-X is restricted such that no user can be closer than 1 m from the perimeter of the system without triggering a radiation interruption. Based on the above initial design assumptions, radiation levels at a wide range of locations 1 m from the circumference of the Zap-X system were measured for all cardinal beam positions. Instantaneous exposure rates, as well as time-integrated measurements of exposures throughout the entire treatment, were determined for 14 equidistant stations around the perimeter of accessible areas of the Zap-X at 1.2 m height above the floor level using a recently calibrated Victoreen Model 451 survey meter (Fluke Biomedical, Everett, Washington, USA). Figure 2 below illustrates the position of measurement locations, as viewed from above. The measurement positions are strictly related to the geometry of the system, as the Monte Carlo-based shielding design was customized to provide identical radiation levels along the 1 m perimeter line.

Radiation shielding as a function of cumulative exposure

Using a reference treatment of 20 Gy, a treatment plan was generated on the Zap-X TPS system and delivered [5]. The cumulative exposure was measured at the same 14 measurement stations around the perimeter of the system as for the exposure rate measurement described above. One additional measurement location atop the system was placed at 1 m above the surface of the treatment sphere (Station #15).

## Results

Instantaneous exposure rates

Instantaneous exposure rates are summarized in Table 1 below, showing the worst-case results at each of the measurement positions, indicating the beam position.

**Table 1:** Summary of instantaneous exposure rates. U indicates the use factor, while D/C is the duty cycle factor of 0.078. The use factor of 0.2 was established by calculating the fraction of time during which a measurement station was exposed to the source of radiation. Gantry position was as indicated. Exposure rates in mR/hr are the worst cases for any of the tested gantry angles.

Location #	Description of Stations	Beam pos.	mR/hr	U	D/C	Ann Dose (mSv)
1	Foot End Table Shield	G = 45	3.0	0.2	0.078	0.936
2	Right Side Main Gantry	G = 45	0.09	0.2	0.078	0.028
3	Left Side Main Gantry	G = 90	0.1	0.2	0.078	0.031
4	Head End of System	G = 45	0.36	0.2	0.078	0.112
5	Table Right	G = 90	0.45	0.2	0.078	0.140
6	Table - Orbit Right	G = 90	0.37	0.2	0.078	0.115
7	Right Gantry	G = 0	0.19	0.2	0.078	0.059
8	Right Gantry	G = 270	1.72	0.2	0.078	0.537
9	Right - Head	G = 45	2.70	0.2	0.078	0.842
10	Left - Head	G = 45	2.80	0.2	0.078	0.874
11	Left Gantry	G = 45	1.70	0.2	0.078	0.530
12	Left Gantry	G = 0	0.57	0.2	0.078	0.178
13	Table - Orbit Left	G = 0	0.84	0.2	0.078	0.262
14	Table Left	G = 0	0.4	0.2	0.078	0.125
15	Control Console	G = 0	0.11	0.2	0.078	0.034

The maximum projected annual dose of 0.936 mSv was determined at the foot end of the table. For 2.7 MV photons with a quality factor of 1.0, exposure values can readily be converted to dose.

Cumulative exposure measurements

For the treatment of plan-produced radiation dose, the cumulative exposure measurements are summarized in Table 2 below. 1.0 mSv is the maximum annual allowable limit for the public, while 50 mSv is the limit for radiation workers. For 2.7 MV accelerating potential, a quality factor of 1.0 was applied. The same assumptions of nine single-isocenter treatment plans with 20 Gy each and 250 working days per year were applied with 2250 annual treatments per year.

**Table 2:** Summary of accumulative exposure measurements.

Station	Exposure (microR)	Annual Dose (mSv)
1	31	0.698
2	5	0.113
3	5	0.113
4	29	0.653
5	32	0.720
6	8	0.180
7	4	0.090
8	25	0.563
9	32	0.720
10	30	0.675
11	24	0.540
12	28	0.630
13	0	0
14	30	0.675
15 atop sphere	55	1.238

The calculated maximum annual exposure was detected at station 15 with 55 microR, corresponding to 1.238 mSv/year. It was assumed that the closest distance a person could be to the top of the treatment sphere, e.g. on the adjacent second floor of a clinic building, would be 1.5 m. Utilizing the above values and applying the inverse-square law reduces the annual cumulative dose to 0.85 mSv at this most superior point. It is acknowledged here that the application of the inverse-square law in reference to the isocenter would somewhat over-estimate the expected annual cumulative dose due to the fact that a distributed source on the surface of the treatment sphere should be assumed. The actual annual cumulative dose would be smaller than 0.85 mSv. The concrete in the floor of the second story would further decrease the dose.

## Discussion

Both the above exposure rates and cumulative dose measurements result in annual dose equivalents below 1.0 mSv/year, which is the generally suggested maximum annual radiation dose for the public, according to the NCRP [1]. In light of this observation, the Zap-X system, under the specified workload, would allow unrestricted access for non-radiation workers outside a 1-m system perimeter and to all areas on the floor above the ZAP treatment room. Therefore, the design of the Zap-X system satisfies all shielding requirements and no shielded “treatment room” is required.

The assumed annual workload of 2250 individual isocentric treatments per year correlates with 1.406 x 10^7^ MU per year being delivered. Based on the experimental results of this study, the maximum allowable workload could be increased slightly, thereby allowing 1.496 x 10^7^ MU per year (2394 individual treatments) while still providing adequate shielding for the public. The acceptable workload can be further increased if the additional occupancy restrictions above and around the treatment space are applied, the area of the treatment space/room is expanded, additional shielding in the form of steel plates is placed at strategic locations, or the surrounding areas are reclassified to be occupationally exposed, thereby requiring radiation badges.

Several benefits are seen to accrue from the reported findings:

1. For a heavy workload, all necessary shielding required for adjacent personnel and the public is integrated as part of the system design, removing the need for construction of a dedicated radiation bunker. As the shielding is provided with the Zap-X, the technical burden of building a dedicated treatment room is removed from the end user institution.

2. Since the shielding requirements for the Zap-X were carefully studied, optimized, and do not depend on a local designer, expected radiation levels outside the treatment sphere are known to meet regulations and safety standards.

3. During clinical implementation, the total required time for facility preparation is decreased by approximately six to 12 months as no treatment bunker construction will be necessary and total construction costs will be reduced significantly.

4. With the above defined heavy workload, expected radiation levels outside the Zap-X shielding are acceptable for the public (1.0 mSv/year), while the typical treatment bunker shielding is designed to protect radiation workers who are allowed to receive a maximum annual dose equivalent of 50 mSv, a factor 50 times higher than the Zap-X.

Exceptionally high treatment workloads can be accommodated by restricting public access in the immediate vicinity, increasing the size of the restricted area, or the addition of minor shielding provisions.

## Conclusions

Experimental analysis supported the hypothesis and demonstrated that a radiosurgical system can be self-shielded, such that it produces radiation exposure levels deemed safe to the public by the NCRP standards while operating under a full clinical workload. The Zap-X has been shown to be a self-shielded radiosurgery system without the need for a radiation treatment bunker at most clinical user sites.
