# Correction: Self-Shielding Analysis of the Zap-X System

**DOI:** 10.7759/cureus.c22

**Published:** 2019-05-17

**Authors:** Georg A. Weidlich, M. Bret Schneider, John R. Adler

**Affiliations:** 1 Radiation Oncology, National Medical Physics and Dosimetry Company, Palo Alto, USA; 2 Department of Neurosurgery, Stanford University School of Medicine, California, USA; 3 Department of Radiation Oncology, Stanford University Medical Center, Stanford, CA, USA; 4 Department of Neurosurgery, Stanford University School of Medicine, Stanford, CA, USA

Patient number 9,757,594 was erroneously included in the Intellectual Property Info section of the Ethics Statement and Conflict of Interest Disclosures. This was a typographical error and the patient number has been removed.

